# Inhibition of Schwann Cell Pyroptosis Promotes Nerve Regeneration in Peripheral Nerve Injury in Rats

**DOI:** 10.1155/2023/9721375

**Published:** 2023-04-25

**Authors:** Jiayi Wang, Shunyi Lu, Ya Yuan, Lei Huang, Mengxuan Bian, Jieqin Yu, Jiapeng Zou, Libo Jiang, Dehua Meng, Jian Zhang

**Affiliations:** ^1^Department of Orthopedic Surgery, Zhongshan Hospital, Fudan University, Shanghai, China; ^2^Department of Rehabilitation, Zhongshan Hospital, Fudan University, Shanghai, China

## Abstract

**Background:**

Peripheral nerve injury (PNI) is one of the most debilitating injuries, but therapies for PNI are still far from satisfactory. Pyroptosis, a recently identified form of cell death, has been demonstrated to participate in different diseases. However, the role of pyroptosis of Schwann cells in PNI remains unclear.

**Methods:**

We established a rat PNI model, and western blotting, transmission electron microscopy, and immunofluorescence staining were used to confirm pyroptosis of Schwann cells in PNI *in vivo*. *In vitro*, pyroptosis of Schwann cells was induced by lipopolysaccharides (LPS)+adenosine triphosphate disodium (ATP). An irreversible inhibitor of pyroptosis, acetyl (Ac)-Tyr-Val-Ala-Asp-chloromethyl ketone (Ac-YVAD-cmk), was used to attenuate Schwann cell pyroptosis. Moreover, the influence of pyroptotic Schwann cells on the function of dorsal root ganglion neurons (DRGns) was analyzed by a coculture system. Finally, the rat PNI model was intraperitoneally treated with Ac-YVAD-cmk to observe the effect of pyroptosis on nerve regeneration and motor function.

**Results:**

Schwann cell pyroptosis was notably observed in the injured sciatic nerve. LPS+ATP treatment effectively induced Schwann cell pyroptosis, which was largely attenuated by Ac-YVAD-cmk. Additionally, pyroptotic Schwann cells inhibited the function of DRGns by secreting inflammatory factors. A decrease in pyroptosis in Schwann cells promoted regeneration of the sciatic nerve and recovery of motor function in rats.

**Conclusion:**

Given the role of Schwann cell pyroptosis in PNI progression, inhibition of Schwann cell pyroptosis might be a potential therapeutic strategy for PNI in the future.

## 1. Introduction

Peripheral nerve injury (PNI) is a common clinical problem that results in substantial disability worldwide [1]. Statistics show that the incidence of PNI ranges from 1.3 to 2.3 per 100,000 people in developed countries, and this incidence may be higher in developing countries [2]. The function of the peripheral nervous system can recover to some extent after PNI, and the extent depends on the severity of the injury and the method of treatment; thus, it is different from the central nervous system [3]. The regenerative process of peripheral nerves is complicated, and the effect of nerve regeneration is always unsatisfactory. The limited treatments for PNI have placed a major burden on social development, yet its underlying mechanisms remain largely unknown.

Schwann cells are a kind of major glial cell in peripheral nervous systems. These cells play a critical role in the regeneration of peripheral nerves, such as the repair of damaged neurons after PNI. After peripheral nerve injury, axons on the distal side of the injured nerve quickly degenerate and disintegrate, and then, debris is phagocytosed and cleared by Schwann cells and macrophages; these phenomena are called Wallerian degeneration [4, 5]. Schwann cells are involved in Wallerian degeneration and other processes of peripheral nerve regeneration [6]. These cells can secrete neurotrophic factors, promote the survival of damaged neurons, and participate in the formation of nerve fibers in the peripheral nervous system. Moreover, Schwann cells can initiate the immune response to recruit monocytes and macrophages to clear myelin debris in the injured sciatic nerve [7]. Therefore, it is important to further explore the roles of Schwann cells in peripheral nerve regeneration.

Pyroptosis is a recently discovered form of programmed lytic cell death. This process is induced by a series of physiological or pathological stimuli, such as glucocorticoids, hypoxia [8], DNA damage [9], and viral infection [10]. These stimuli result in mitochondrial damage and an increase in reactive oxygen species (ROS) [11]. In the latter, NOD-like receptor family pyrin domain-containing 3 (NLRP3) inflammatory bodies interact with apoptosis-associated speck-like protein containing CARD (ASC) and the effector molecule cysteinyl aspartate-specific protease-1 precursor (pro-caspase-1), resulting in the formation of inflammasome complexes and Cleaved-casp-1 [12, 13]. Gasdermin D (GSDMD) and the precursor inflammatory factors pro-IL-18 and pro-IL-1*β* were then cleaved by Cleaved-casp-1, the pivotal mediating factor for pyroptosis. GSDMD is composed of the N-terminal fragment of GSDMD (N-GSDMD) and the C-terminal fragment of GSDMD (C-GSDMD). After cleavage by Cleaved-casp-1, N-GSDMD oligomerizes in the cytomembrane to form pores that increase cell membrane permeability. Subsequently, IL-18 and IL-1*β* are released from the pores in the cytomembrane generated by N-GSDMD [14]. Recent studies have verified that pyroptosis is involved in the pathological process of many diseases, such as septicemia [15], diabetic cardiomyopathy [16], atherosclerosis [17, 18], various tissue injuries [19, 20], and even Alzheimer's disease [21]. Moreover, researchers have found that macrophage pyroptosis is involved in PNI in mice [22]. However, the role of Schwann cell pyroptosis in PNI is still unknown.

In this study, we explored the role of Schwann cell pyroptosis in PNI. We investigated the ability of LPS+ATP to induce Schwann cell pyroptosis and Ac-YVAD-cmk to suppress pyroptosis. Then, we explored the effect of Schwann cell pyroptosis on the function of surrounding neurons. Moreover, the effect of Ac-YVAD-cmk on peripheral nerve regeneration was observed *in vivo*.

## 2. Methods

### 2.1. Ethics Statement and Animals

Six- to eight-week-old male Sprague-Dawley rats (200-250 g) were housed in temperature- and humidity-controlled quarters in the Lab Animal Center of Zhongshan Hospital, Fudan University. The study was approved by the ethics committee of Zhongshan Hospital, and all experimental procedures were approved by the Animal Care and Use Committee of Zhongshan Hospital.

### 2.2. Animal Experiments

Animal experiment was divided into two parts in the study; the total number of rats was 180. We first confirmed Schwann pyroptosis exist in PNI, the total number of rats was 126, and the rats were divided into seven groups (*n* = 18). Then, the rat PNI model was intraperitoneally treated with Ac-YVAD-cmk to observe the effect of pyroptosis on nerve regeneration, the total number of rats was 54, and the rats were divided into three groups (*n* = 18).

#### 2.2.1. PNI Model

Rats were randomly divided into groups as follows: sham-operation controls (control, *n* = 18) and the PNI group (*n* = 108) at 0.5, 1, 2, 3, 7, and 14 d (*n* = 18 for each observation time point). All rats were anesthetized and kept on a heating pad during anesthesia. The left hind leg of each rat was prepared for surgery after shaving and disinfection. With aseptic techniques, the sciatic nerve was isolated and transected in a transverse incision below the femur in the PNI group, and then, the proximal and distal nerve stumps were sutured under a microscope [23]. The sciatic nerve was exposed without damage, and then, the muscle layers and skin were sutured in the control group. The nerve samples 1 cm distal to the nerve transection site were collected separately from rats after transection in the PNI group, and nerve samples of equal length were also collected in the control group. All the procedures were performed by the same investigator.

#### 2.2.2. Regeneration of Peripheral Nerve

The rat PNI model was intraperitoneally treated with Ac-YVAD-cmk to observe the effect of pyroptosis on nerve regeneration. A total of 54 rats were randomly divided into three groups as follows: Group 1: sham operation (control, *n* = 18); Group 2: sciatic nerve transection with immediate repair followed by intraperitoneal administration of placebo (PNI, *n* = 18); Group 3: sciatic nerve transection with immediate repair followed by intraperitoneal administration of Ac-YVAD-cmk (PNI+AC, *n* = 18). Ac-YVAD-cmk (25 mg/kg) or an equal volume of placebo was administered daily for 7 days after surgery [24, 25]. Sciatic nerve segments 1 cm distal to the nerve transected site were taken on the 7th postoperative day for western blotting and immunofluorescence staining analysis and on the 90th postoperative day for transmission electron microscope analysis. All the procedures were performed by the same investigator.

### 2.3. Antibodies

Caspase-1 (Lot: #ab179515), NLRP3 (Lot: #ab263899), and S100 antibodies (Lot: #ab52642) were purchased from Abcam (Cambridge, England, UK). GSDMD antibody (Lot: #E9S1X) and *β*-actin antibody (Lot: #4967) for western blotting were purchased from Cell Signaling Technology (Massachusetts, USA). The GSDMD (Lot: #20770-1-AP) and *β*III-Tubulin (Lot: #66375-1-Ig) primary antibodies for immunofluorescence were purchased from Proteintech (Wuhan, China). The NeuN primary antibody (Lot: #GB11138) was obtained from Servicebio (Wuhan, China). The rat anti-IL-18 antibody (Lot: #CZU0220031) and anti-IL-1*β* antibody (Lot: #YR0920081) were obtained from R&D Systems (Minneapolis, MN, USA).

### 2.4. Cell Line and Culture Conditions

RSC96 cells were obtained from the Cell Bank of the Chinese Academy of Sciences and cultured in Dulbecco's modified Eagle's medium (DMEM, Gibco) with 10% fetal bovine serum (FBS, Gibco) and 1% antibiotics (100 *μ*g/ml streptomycin and 100 *μ*g/ml penicillin).

For the Schwann cell pyroptosis model *in vitro*, different concentrations (0.1, 1 *μ*g/ml) of LPS (Lot: #L4391-1MG, Sigma-Aldrich, USA) were used to stimulate RSC96 cells for 4 h, and then, these cells were treated with various concentrations (2.5, 5 mM) of ATP (Lot: #A2383-5G, Sigma-Aldrich) for the indicated times (1, 6 h). The caspase-1 inhibitor Ac-YVAD-cmk (Lot: #SML0429, Sigma-Aldrich) was added to Schwann cells 2 h before LPS stimulation [26, 27].

### 2.5. Dorsal Root Ganglia Neuron (DRGn) Cultures

Purified primary DRGns were derived from the L1-L6 DRG of adult male Sprague-Dawley rats (200-250 g) as described previously [28, 29]. DRGns were maintained with 50 ng/ml nerve growth factor (NGF; Lot: #450-34, PeproTech, USA) in 1% N2-supplemented (Lot: #17502048, Gibco) medium for 1 week before coculture with Schwann cells.

### 2.6. Cell Viability Assays

The Cell Counting Kit-8 assay (CCK8; Beyotime, Shanghai, China) was used to evaluate the viability of Schwann cells according to the manufacturer's protocol. Schwann cells were seeded in a 96-well plate at 3 × 10^4^ cells/well overnight. The optical density (OD) values at 450 nm were measured using a microplate reader (Bio-Rad, Hercules, USA) at each time point.

### 2.7. Immunofluorescence Staining

After the rats were anesthetized, sciatic nerve segments 1 cm distal to the nerve transection site were collected and fixed in embedding medium. Then, the nerve slides were permeabilized with 0.3% Triton X-100; after 3 washes with PBS, the sections were blocked in 5% bovine serum albumin (BSA) for 1 h. The sections were then incubated with primary antibodies against GSDMD (1 : 1000) and S100 (1 : 1000) overnight at 4°C. Next, after three washes with PBS, the slides were incubated with goat secondary antibodies for 1 h. Finally, the sections were covered with 4′,6-diamidino-2-phenylindole (DAPI; Lot: #28718-90-3, Sigma-Aldrich). Images were visualized and captured using a fluorescence microscope (Nikon, Tokyo, Japan).

For the experimental part of cell immunofluorescence staining, briefly, Schwann cells after different treatments were incubated with primary antibodies GSDMD (1 : 1000) and a cell membrane fluorescent probe, DiI (1,1′-dioctadecyl-3,3,3′,3′-tetramethylindocarbocyanine perchlorate), which was purchased from Beyotime (Lot: #C1991S). Nuclei were stained with DAPI. After coculture with different groups of Schwann cells, primary DRGns were incubated with primary antibodies against NeuN (1 : 500) and *β*III-Tubulin (1 : 1000). Nuclei were stained with DAPI.

### 2.8. Western Blotting

Total protein was extracted from sciatic nerve samples, cultured Schwann cells or using RIPA buffer (Lot: #P0013B, Beyotime). Twenty-five milligrams of protein was resolved by SDS-PAGE and then transferred to polyvinylidene fluoride (PVDF) membranes. PVDF membranes were incubated with primary antibodies overnight at 4°C. Then, the cells were incubated with secondary antibodies for 1 h at room temperature. Finally, proteins were visualized using a chemiluminescent peroxidase substrate.

### 2.9. Enzyme-Linked Immunosorbent Assay (ELISA)

The culture supernatant of treated cells was harvested, and the levels of IL-18 (Lot: #E-EL-R0567c, Elabscience, China) and IL-1*β* (Lot: #E-EL-R0012c, Elabscience) were detected by ELISA kits following the manufacturer's protocols.

### 2.10. Real-Time PCR

Total RNA was extracted using TRIzol reagent (Lot: #R0016, Beyotime), and cDNA was obtained after reverse transcription with a PrimeScript RT Reagent Kit (Lot: #RR047A, TaKaRa, Japan). Real-time PCR was performed with a One-Step RT-qPCR SYBR Green Kit (Lot: #11143ES50, Yeasen, Shanghai, China) via an ABI System. The sequences of the IL-6 primers used to produce the desired PCR product were 5′-CTTCCCTACTTCACAAGTC-3′ (forward primer) and 5′-CTCCATTAGGAGAGCATTG-3′ (reverse primer). The sequences of the TNF-*α* primers used were 5′-CTCAGCCTCTTCTCACTTCC-3′ (forward primer) and 5′-GCAGAGAGGAGGTTGACTC-3′ (reverse primer). The sequences of the *β*-actin primers used were 5′-CATGTACGTTGCTATCCAGGC-3′ (forward primer) and 5′-CTCCTTAATGTCACGCACGAT-3′ (reverse primer).

### 2.11. Transmission Electron Microscopy

Transmission electron microscopy was used to verify the morphology of pyroptotic Schwann cells in the injured sciatic nerve. The ball-like bulge morphology of pyroptosis in the injured sciatic nerve was observed by transmission electron microscopy at different time points after injury, and representative images are shown in this study.

In the Ac-YVAD-cmk treatment experimental section *in vivo*, the electron micrographs from six random fields (×1000 magnification) of each group were observed, and the average myelinated axon diameter and thickness of the myelin sheath were assessed based on the electron micrographs by using ImageJ software [30, 31].

### 2.12. Coculture System

Transwell chamber systems were constructed to investigate the effect of pyroptotic Schwann cells on the function of DRGns [32]. Schwann cells were seeded in the upper chamber, and DRGns were seeded in the lower chamber. DRGns were cultured in a 24-well plate for 1 week before the transwell cell insert (0.4 *μ*m pore size, Corning) containing Schwann cells (1 × 10^4^) that were treated with LPS+ATP+PBS, LPS+ATP+Ac-YVAD-cmk (AC), or LPS+ATP+neutralizing antibody (NA) was added to each well of the plate. The control group is the DRGns cocultured with Schwann cells without any treatment. The transwell cell insert was washed three times with PBS before being added to the well of the plate to eliminate the effect of residual ATP on inferior ventricular DRGns. After 24 h of coculture, DRGns were collected for immunofluorescence staining or PCR assays. For immunofluorescence staining, the average body size and axonal length of DRGns were analyzed using Image-Pro-Plus (SIS, Münster, Germany).

### 2.13. Walking Track Analysis

The sciatic functional index (SFI) is generally used to assess behavioral recovery and restoration of function after sciatic nerve injury [33]. SFI was performed to assess sciatic behavioral function at different time points after surgery in this study. Briefly, hind paws were dipped in black ink, and rats of different groups were allowed to walk down a narrow channel, making footprints on a white paper. SFI was calculated according to the following equation: SFI = −38.3 × (EPL − NPL)/NPL + 109.5 × (ETS − NTS)/NTS + 13.3 × (EIT − NIT)/NIT − 8.8. PL is the distance from the heel to the third toe, TS is the distance from the first to the fifth toe, and IT is the distance from the second to the fourth toe. E and N represent the experimental and normal groups, respectively.

### 2.14. Statistical Analysis

Statistical analyses were analyzed using GraphPad Prism 8.0 software. One-way ANOVA was used to evaluate the differences among the different groups. Statistical analyses were performed with SPSS 23.0 software (SPSS, Inc., Chicago, IL, USA). *p* < 0.05 was considered statistically significant.

## 3. Results

### 3.1. PNI Induces Schwann Cell Pyroptosis In Vivo

**Figure 1:**
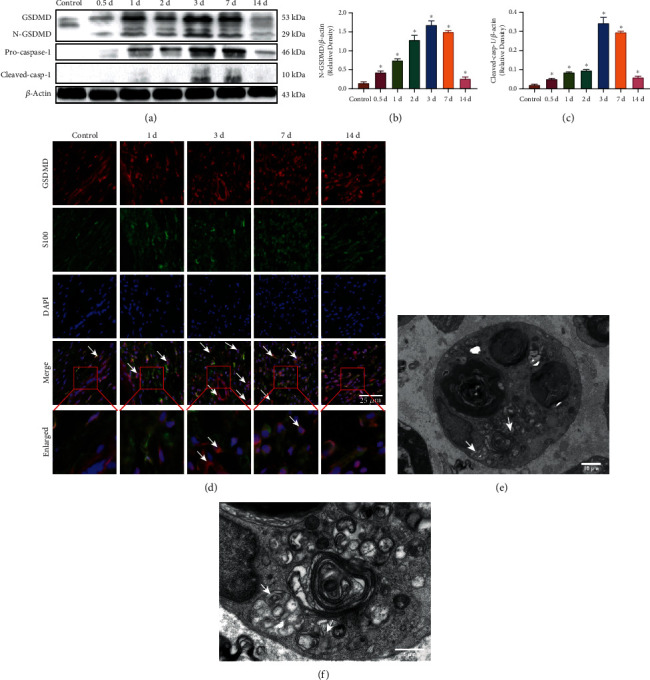
Injury induced Schwann cell pyroptosis in peripheral nerves *in vivo*. (a) The levels of pyroptosis-associated proteins were detected by western blot analysis in the injured sciatic nerve after transection. *β*-Actin was used as an internal control. (b, c) Levels of N-GSDMD and Cleaved-casp-1 were quantified by densitometric measurement. ^∗^*p* < 0.05 compared with the control group. Data are expressed as the mean ± SEM. All experiments were repeated three times. (d) Representative images of immunofluorescence staining of GSDMD (red) and S100 (green) with DAPI (blue) in sciatic nerve sections after transection. Cells with GSDMD, S100, and DAPI expression (yellow) are labeled with arrowheads. Scale bar, 25 *μ*m. (e, f) Transmission electron microscope observation of the injured sciatic nerve on the 3rd postoperative day. Red arrowhead indicates Schwann cell nucleus pyknosis, and white arrowheads indicate large bubbles emerging from the cytoplasm of Schwann cells. Scale bars: (e) 10 *μ*m and (f) 5 *μ*m.

We first verified whether pyroptosis exists in Schwann cells in the PNI rat model using western blotting, immunofluorescence staining, and transmission electron microscopy. The levels of pyroptosis-associated proteins in the sciatic nerve were evaluated by western blotting after PNI at different time points after surgery. Compared with those of the control group, the expression levels of N-GSDMD and Cleaved-casp-1 were notably upregulated in transected rat sciatic nerve tissue and peaked on the 3rd day in the PNI model (Figures 1(a)–1(c)). In addition, GSDMD colocalization with S100 peaked on the 3rd day after PNI (Figure 1(d)), which was consistent with the western blot results, suggesting that high levels of GSDMD were expressed in Schwann cells on the 3rd postoperative day. Furthermore, ball-like bulges were observed in damaged Schwann cells, especially on the 3rd day (Figures 1(e) and 1(f)). These results suggested that PNI induced Schwann cell pyroptosis in rats.

### 3.2. LPS+ATP Induce Pyroptosis in Schwann Cells

**Figure 2:**
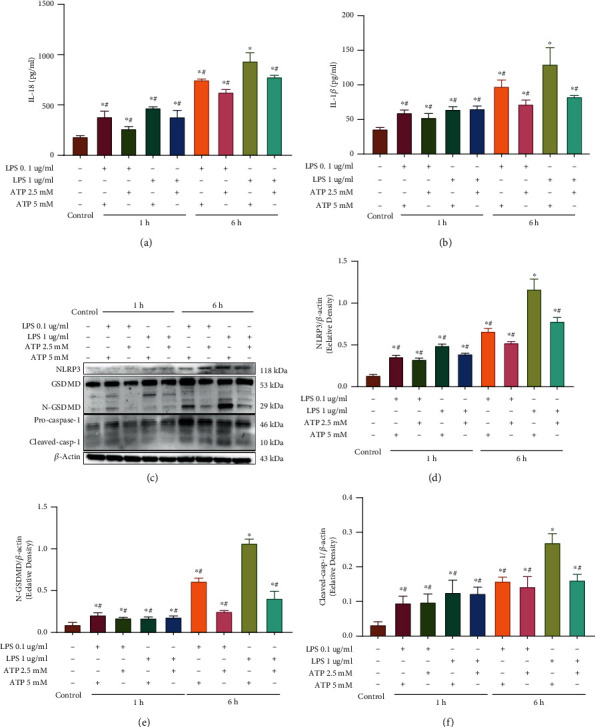
LPS+ATP induced pyroptosis of Schwann cells *in vitro*. (a, b) IL-18 and IL-1*β* levels in the culture supernatant determined by ELISAs. ^∗^*p* < 0.05 compared with the control group. ^#^*p* < 0.05 compared with the 1 *μ*g/ml LPS+5 mM ATP (6 h) group. Data are expressed as the mean ± SEM. (c) Western blot analysis of N-GSDMD, NLRP3, and Cleaved-casp-1 in the Schwann cells treated with the indicated doses and times of LPS and ATP. *β*-Actin was used as an internal control. (d–f) Semiquantitative analysis of the levels of N-GSDMD, NLRP3, and Cleaved-casp-1 in western blotting. *β*-Actin was used as an internal control. ^∗^*p* < 0.05 compared with the control group. ^#^*p* < 0.05 compared with the 1 *μ*g/ml LPS+5 mM ATP (6 h) group. Data are expressed as the mean ± SEM. All experiments were repeated three times.

To further confirm the phenotype of pyroptosis in Schwann cells, we treated these cells with different concentrations of LPS (0.1, 1 *μ*g/ml) and ATP (2.5, 5 mM) to induce pyroptosis, except for the control group. Previous studies revealed that the inflammatory response was linked to pyroptosis [34]; thus, we wondered whether combining LPS with ATP induced an inflammatory response in Schwann cells. The ELISA results revealed that the inflammatory cytokines IL-18 and IL-1*β* in the culture supernatant increased and peaked in the 1 *μ*g/ml LPS+5 mM ATP (6 h) group after LPS+ATP treatment (Figures 2(a) and 2(b)). Moreover, the western blotting results demonstrated that the protein levels of NLRP3, N-GSDMD, and Cleaved-casp-1 were significantly elevated and peaked in the 1 *μ*g/ml LPS+5 mM ATP (6 h) group (Figures 2(c)–2(f)) compared to the control group. Herein, the above data suggested that combining LPS with ATP could induce pyroptosis in Schwann cells, and a 1 *μ*g/ml LPS+5 mM ATP (6 h) model was used to induce pyroptosis in subsequent experiments.

### 3.3. Ac-YVAD-cmk (AC) Attenuates Pyroptosis Induced by LPS+ATP in Schwann Cells

**Figure 3:**
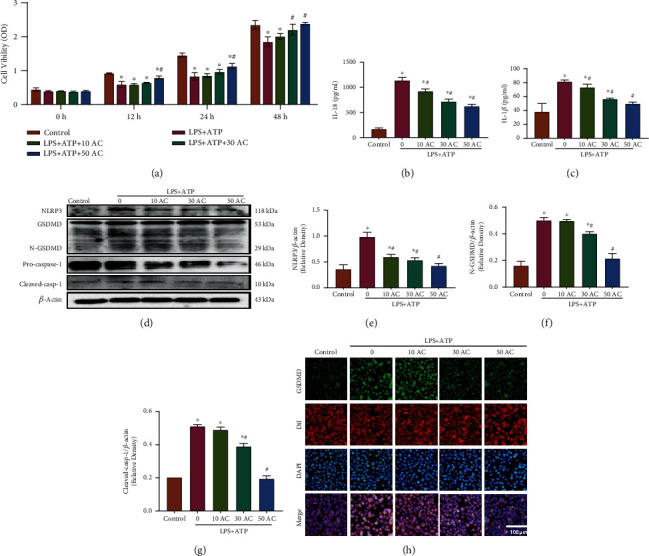
Ac-YVAD-cmk attenuated Schwann cell pyroptosis. (a) A CCK-8 assay was used to measure the viability of Schwann cells treated with various concentrations of Ac-YVAD-cmk (10, 30, and 50 *μ*M). ^∗^*p* < 0.05 compared with the control group. ^#^*p* < 0.05 compared with the LPS+ATP group. Data are expressed as the mean ± SEM. (b, c) IL-18 and IL-1*β* levels in the culture supernatant determined by ELISAs. ^∗^*p* < 0.05 compared with the control group. ^#^*p* < 0.05 compared with the LPS+ATP group. Data are expressed as the mean ± SEM. (d) NLRP3, N-GSDMD, and Cleaved-casp-1 levels were detected by western blot analysis. (e–g) Levels of NLRP3, N-GSDMD, and Cleaved-casp-1 were quantified by densitometric measurement. ^∗^*p* < 0.05 compared with the control group. ^#^*p* < 0.05 compared with the LPS+ATP group. Data are expressed as the mean ± SEM. (h) Schwann cells were incubated with GSDMD and DiI after treatment. Immunofluorescence analysis of the distribution of GSDMD in the membranes of Schwann cells induced by LPS and ATP treatment and then exposed to different concentrations of the pyroptosis inhibitor Ac-YVAD-cmk (10, 30, and 50 *μ*M). Scale bar, 100 *μ*m.

To further confirm pyroptosis in Schwann cells, we used the caspase-1 inhibitor Ac-YVAD-cmk to attenuate LPS+ATP-induced pyroptosis in Schwann cells [35]. The CCK-8 assay showed that the cell viability of the 50 *μ*M AC/LPS+ATP group was higher at 12, 24, and 48 h than that of the LPS+ATP group, while it was still lower than that of the control group (*p* < 0.05, Figure 3(a)). We also found that the levels of IL-18 and IL-1*β* in the 50 *μ*M AC/LPS+ATP group were obviously downregulated (*p* < 0.05, Figures 3(b) and 3(c)). Furthermore, the protein levels of NLRP3, N-GSDMD, and Cleaved-casp-1 were significantly reduced compared with those in the LPS+ATP group (Figures 3(d)–3(g)). Finally, GSDMD was colocalized with DiI, a cell membrane fluorescent probe (Figure 3(h)). The levels of GSDMD in the LPS+ATP group were significantly higher than those in the control group in both the cytoplasm and membrane of Schwann cells. The mean fluorescence intensity (MFI) of GSDMD at the surface and cytoplasm was decreased in the LPS+ATP+50 AC group compared to the LPS+ATP group. According to the above results, 50 *μ*M Ac-YVAD-cmk was used to inhibit pyroptosis in the following study.

### 3.4. Pyroptosis of Schwann Cells Inhibits the Function of Cocultured DRGns

**Figure 4:**
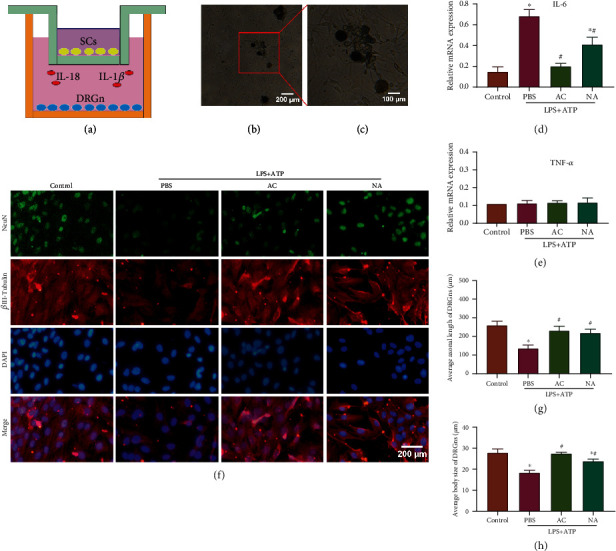
Schwann cell pyroptosis suppressed the function of cocultured DRGns. (a) Schematic for the coculture procedure. (b, c) A representative ordinary optics microscope image of primary DRGns before coculture with Schwann cells. Scale bar, (b) 200 *μ*m and (c) 100 *μ*m. Real-time PCR was performed to detect the mRNA expression of (d) IL-6 and (e) TNF-*α*. ^∗^*p* < 0.05 compared with the control group. ^#^*p* < 0.05 compared with the LPS+ATP+PBS group. (f) Immunofluorescence analysis of the distribution of NeuN and *β*III-Tubulin in DRGns after 24 h of coculture with different groups of Schwann cells, and the nuclei were counterstained using DAPI. Scale bar, 200 *μ*m. (g, h) The average axonal length and body size of DRGns cocultured with different groups of Schwann cells were analyzed. ^∗^*p* < 0.05 compared with the control group. ^#^*p* < 0.05 compared with the LPS+ATP+PBS group. Data are expressed as the mean ± SEM.

Schwann cells and DRGns were cocultured using the transwell system (Figure 4(a)). The representative morphology of primary DRGns before coculture with Schwann cells on the 7th day after extraction is shown at different magnifications (Figures 4(b) and 4(c)). Real-time PCR results showed that the gene expression of IL-6 in DRGns in the LPS+ATP+PBS group and the LPS+ATP+NA group was significantly higher than that in the control group (*p* < 0.05), and the gene expression of IL-6 in DRGns in the LPS+ATP+AC group and the LPS+ATP+NA group was significantly lower than that in the LPS+ATP+PBS group (Figure 4(d)). There was no significant difference in the expression of TNF-*α* (Figure 4(e)). Immunofluorescence staining was used to detect the location of specific markers of neurons, *β*III-Tubulin and NeuN, of DRGns (Figure 4(f)) to evaluate the function of DRGns. The MFI of *β*III-Tubulin and NeuN in the LPS+ATP+PBS group was significantly lower than that in the LPS+ATP+AC group and the LPS+ATP+NA group (*p* < 0.05), and there was no significant difference between the control group and the LPS+ATP+AC group. The average body size and axonal length of DRGns cocultured with Schwann cells from different groups were analyzed. The average body size and axonal length of DRGns in the LPS+ATP+PBS group were significantly lower than those in the three other groups, and there was no significant difference between the control group and the LPS+ATP+AC group (Figures 4(g)–4(h)). Taken together, these data proved that pyroptosis of Schwann cells inhibited the function of cocultured DRGns and that Ac-YVAD-cmk could attenuate the inhibitory effects of pyroptotic Schwann cells on cocultured DRGns.

### 3.5. Ac-YVAD-cmk Promotes the Regeneration of Peripheral Nerves by Inhibiting Schwann Cell Pyroptosis In Vivo

**Figure 5:**
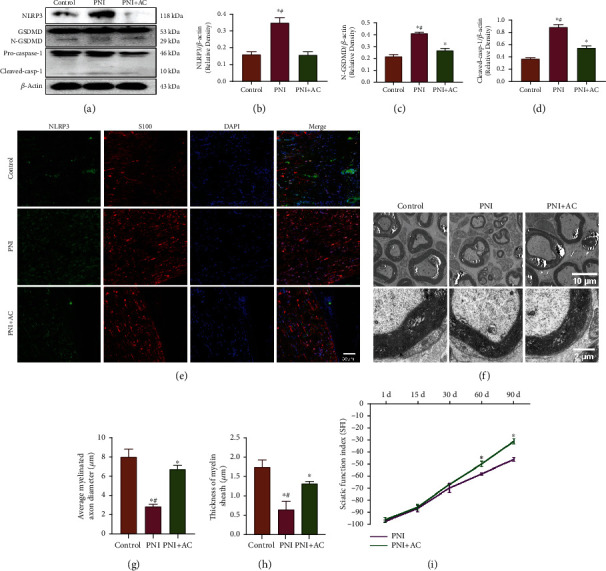
Ac-YVAD-cmk promotes nerve regeneration in peripheral nerve injury. (a) NLRP3, N-GSDMD, and Cleaved-casp-1 levels were detected by western blot analysis. *β*-Actin was used as an internal control. (b–d) Levels of N-GSDMD, NLRP3, and Cleaved-casp-1 were quantified by densitometric measurement. ^∗^*p* < 0.05 compared with the control group. ^#^*p* < 0.05 compared with the LPS+ATP+AC group. Data are expressed as the mean ± SEM. (e) Immunofluorescence analysis of the distribution of NLRP3 and S100 in sciatic nerve sections. Scale bar, 50 *μ*m. (f) Representative transmission electron microscopic images were selected from three different groups. Scale bars, 10 *μ*m and 2 *μ*m. (g, h) Statistical results of the average myelinated axon diameter and thickness of the myelin sheath in rats from different groups. ^∗^*p* < 0.05 compared with the control group. ^#^*p* < 0.05 compared with the LPS+ATP+AC group. Data are expressed as the mean ± SEM. (i) The sciatic function index (SFI) values in all groups were measured at the predetermined time postoperatively.

Ac-YVAD-cmk protected against injury-induced Schwann cell pyroptosis in peripheral nerves by inhibiting Cleaved-casp-1-mediated pyroptotic cell death through downregulation of the protein levels of NLRP3 and N-GSDMD, as well as attenuating pro-IL-1*β* and pro-IL-18, which were cleaved into mature IL-1*β* and IL-18. Measurement of the levels of the pyroptosis-associated proteins NLRP3, N-GSDMD, and Cleaved-casp-1 revealed that injury significantly induced Schwann cell pyroptosis in the PNI model (Figures 5(a)–5(e)). Nevertheless, Ac-YVAD-cmk treatment dramatically attenuated the pyroptosis-associated protein levels in the PNI+AC group compared with the PNI group (*p* < 0.05). These aforementioned results proved that Ac-YVAD-cmk could suppress injury-induced Schwann cell pyroptosis in the sciatic nerve.

Transmission electron microscopy was used to observe the morphological features of peripheral nerves in different groups on the 90th postoperative day (Figure 5(f)). The average myelinated axon diameter and thickness of the myelin sheath of regenerated nerve fibers in the PNI+AC group were significantly better than those in the PNI group, while they were still lower than those in the control group (*p* < 0.05, Figures 5(g) and 5(h)).

**Figure 6:**
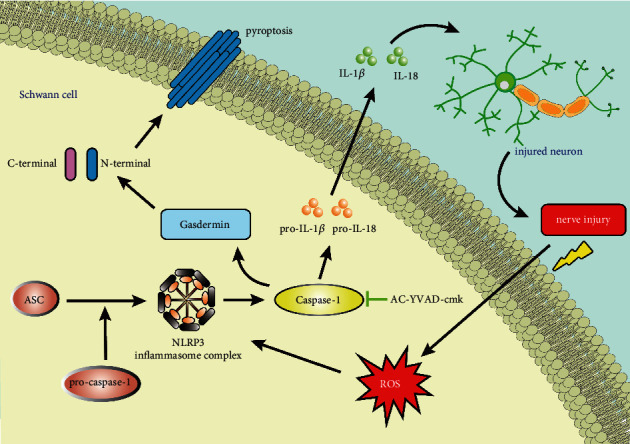
Schematic of the protective effect of the pyroptosis inhibitor Ac-YVAD-cmk on peripheral nerve injury.

To assess the possible effects of Ac-YVAD-cmk on motor functional recovery, we determined the SFI to evaluate the recovery of motor function after sciatic nerve injury in rats. The SFI value varies from −100 to 0, with −100 and 0 indicating absolute loss and normal motor function of the sciatic nerve, respectively. No significant difference in the SFI value was observed among rats in different groups on the 1st, 15th, and 30th postoperative day, but the SFI value was significantly higher in the rats treated with Ac-YVAD-cmk at other time points after surgery than in the rats treated with placebo (*p* < 0.05, Figure 5(i)). Given the crucial role of Schwann cells in the regeneration of peripheral nerves, these results suggest that Ac-YVAD-cmk can promote nerve regeneration in peripheral nerve injury by attenuating pyroptosis of Schwann cells in rats. The schematic of the protective effect of the pyroptosis inhibitor Ac-YVAD-cmk on peripheral nerve injury by targeting Caspase-1 is shown in Figure 6.

## 4. Discussion

In this study, we established a peripheral nerve injury model and confirmed that pyroptosis is a major type of Schwann cell death. We confirmed that LPS+ATP treatment could effectively induce pyroptosis of Schwann cells, which could be attenuated by the pyroptosis inhibitor Ac-YVAD-cmk. Pyroptosis of Schwann cells can lead to dysfunction of DRGns by the inflammatory mediators IL-1*β* and IL-18. Most importantly, Ac-YVAD-cmk was shown to promote nerve regeneration in peripheral nerve injury by attenuating pyroptosis of Schwann cells in rats.

Complete transection injury is usually used as a model for the study of PNI [22], and we used transection injuries as a model in this study. In the present study, transection injury released many danger-associated molecular patterns (DAMPs) [36], which induced subsequent Schwann cell pyroptosis. Herein, pyroptotic Schwann cells were found in the PNI rat model, especially on the 3rd postoperative day. Pyroptosis of Schwann cells was attenuated on the 7th and 14th postoperative day. We speculated that many DAMPs were gradually released from damaged tissues and accumulated at the surrounding Schwann cells, and these cytokines and mediators induced Schwann cell pyroptosis [37]. Then, the number of pyroptotic Schwann cells was reduced along with the repair of tissue damage [36]. Thus, these data suggested that DAMPs induced Schwann cell pyroptosis.

LPS+ATP treatment is commonly used to induce cell pyroptosis *in vitro* [38–41]. Consistent with previous findings, LPS combined with ATP elevated the levels of the pyroptosis-associated proteins NLRP3, N-GSDMD, and Cleaved-casp-1. The caspase-1 inhibitor Ac-YVAD-cmk dramatically inhibited pyroptosis induced by LPS+ATP in Schwann cells. These data revealed that pyroptosis in the Schwann cell model was established using LPS+ATP treatment.

Schwann cells have positive effects on the function of neuronal cells [42, 43]; however, few studies have reported the effects of pyroptotic Schwann cells. Herein, we found that pyroptotic Schwann cells suppressed the function of neurons and promoted the inflammatory response. However, inhibition of pyroptosis in Schwann cells improved neuron function and attenuated inflammatory mediator production in peripheral nerves. Pyroptosis of cells can release many inflammatory mediators, such as IL-1*β* and IL-18, and these mediators further induce pyroptosis in the surrounding cells, which ultimately exacerbates tissue damage. Previous studies proved that IL-1*β* and IL-18 induced nerve injury and impaired the function of neurons [42, 43]. Our study revealed that Schwann cell pyroptosis released IL-1*β* and IL-18. Further analysis showed that downregulation of inflammatory cytokines using a neutralizing antibody also improved the function of DRGns. Together, these data suggest that pyroptosis of Schwann cells causes dysfunction of dorsal root ganglion neurons through the release of inflammatory cytokines.

Ac-YVAD-cmk is usually used to inhibit pyroptosis by intraperitoneal injection *in vivo* [35, 44]. Next, we validated the effect of Ac-YVAD-cmk in the regeneration of peripheral nerves. Levels of pyroptosis-associated proteins were found to be dramatically downregulated in the PNI+AC group compared with the PNI group, which proves that Ac-YVAD-cmk is effective in inhibiting pyroptosis of Schwann cells. The morphological features of regenerated nerve fibers can reflect the degree of functional recovery after peripheral nerve injury [45]. In the present study, after treatment with Ac-YVAD-cmk, the average myelinated axon diameter and thickness of the myelin sheath were significantly improved in regenerated nerve fibers after sciatic injury. The SFI value is a reliable index for detecting motor function recovery of injured sciatic nerves. Administration of Ac-YVAD-cmk by intraperitoneal injection in rats caused a noticeable acceleration in the recovery of motor function from the 60th day after surgery. The above evidence proves that Ac-YVAD-cmk can effectively promote peripheral nerve regeneration by attenuating pyroptosis of Schwann cells in rats.

## 5. Conclusion

Our studies provide novel insight into one of the mechanisms of Schwann cells in peripheral nerve regeneration. We demonstrate that pyroptosis of Schwann cells induced by peripheral nerve injury impaired peripheral nerve regeneration by releasing inflammatory cytokines, which could be attenuated by the pyroptosis inhibitor Ac-YVAD-cmk. More importantly, inhibition of pyroptosis in Schwann cells may reduce the inflammatory response and promote nerve regeneration in peripheral nerve injury, which provides a potential therapeutic strategy in the future.

## Data Availability

All data supporting the findings of this report are included in this article. Source raw data for all quantitations is fully available upon request.
